# Patient satisfaction with divided anesthesia care

**DOI:** 10.1007/s00101-022-01192-x

**Published:** 2022-08-29

**Authors:** Kira-Lee Koster, Carolin Björklund, Sebastian Fenner, Wolfgang Johann Flierler, Michael Laupheimer, Katharina Burri, Matthias Nübling, Thomas Heidegger

**Affiliations:** 1 Department of Anaesthesia, Spital Grabs, Spitalstr. 44, 9472 Grabs, Switzerland; 2 Institute of Anaesthesiology, Intensive Care and Pain Therapy, Klinikum Memmingen, Memmingen, Germany; 3 Institute of Anaesthesiology and Intensive Care, Hirslanden Klinik Stephanshorn, St. Gallen, Switzerland; 4 Data and Consulting, GDB mbH, Denzlingen, Germany; 5 Department of Anaesthesiology and Pain Medicine, Bern University Hospital, University of Bern, Bern, Switzerland; 6 Department of Medical Oncology and Haematology, Cantonal Hospital St. Gallen, St. Gallen, Switzerland

**Keywords:** Anesthesia, Quality, Patient satisfaction, Psychometrically developed questionnaire, Divided anesthesia care, Anästhesie, Qualität, Patientenzufriedenheit, Psychometrisch entwickelter Fragebogen, Geteilte Anästhesiebetreuung

## Abstract

**Background:**

Up to now, no prospective cohort study using a validated questionnaire has assessed patients’ expectation and perception of divided anesthesia care and its influence on patient satisfaction.

**Objective:**

We assessed patient satisfaction with divided anesthesia care in a district general hospital in Switzerland. We hypothesized that patient expectations, combined with their perceptions of the (un)importance of continuous anesthesia care would influence patient satisfaction.

**Material and methods:**

A total of 484 eligible in-patients receiving anesthesia from October 2019 to February 2020 were included and received preoperative information about divided care via a brochure and face-to-face. The primary outcome was the assessment of patient satisfaction with divided anesthesia care using a validated questionnaire. In group 1 continuity of care was considered important but not performed. In group 2 continuity was ensured. In group 3 continuity was regarded as not important and was not performed. In group 4 patients could not remember or did not answer. A psychometrically developed validated questionnaire was sent to patients at home after discharge.

**Results:**

A total of 484 completed questionnaires (response rate 81%) were analyzed. In group 1 (*n* = 110) the mean total dissatisfaction score was 25% (95% confidence interval [CI] 21.8–28.1), in group 2 (*n* = 61) 6.8% (95% CI 4.8–8.7), in group 3 (*n* = 223) 12.1% (95% CI 10.7–13.4), and in group 4 (*n* = 90) 15% (95% CI 11–18); ANOVA: *p* < 0.001, η = 0.43. Of the patients 286 (59%) considered continuity of care by the same anesthetist relatively unimportant (34%) or not important at all (25%). The other 40% considered it important (22%) or very important (18%).

**Conclusion:**

Despite receiving comprehensive preoperative information about divided anesthesia care, 40% of patients still considered continuity of care by the same anesthetist important. We recommend further research evaluating whether and how patient expectations can be modified towards the common practice of divided care and patient satisfaction can be increased.

**Supplementary Information:**

Die Online-Version dieses Beitrags (10.1007/s00101-022-01192-x) enthält elektronisches Zusatzmaterial. Beitrag und Zusatzmaterial stehen Ihnen auf www.springermedizin.de zur Verfügung. Bitte geben Sie dort den Beitragstitel in die Suche ein, das Zusatzmaterial finden Sie beim Beitrag unter „Ergänzende Inhalte“.

## Abstract (de)

**Hintergrund:**

Bis heute gibt es keine prospektive Kohortenstudie, die mittels eines validierten Fragebogens die Erwartung und Wahrnehmung einer geteilten Anästhesiebetreuung („divided care“) und deren Einfluss auf die Patientenzufriedenheit erhoben hat.

**Ziel der Arbeit:**

Es wurde die Hypothese aufgestellt, dass die Patientenerwartung, kombiniert mit der Wahrnehmung der (Un)wichtigkeit der kontinuierlichen anästhesiologischen Patientenbetreuung (präoperatives Informations- und Aufklärungsgespräch und Durchführung der Anästhesie durch die gleiche Person) einen Einfluss auf die Zufriedenheit habe.

**Material und Methoden:**

In einem mittelgroßen Schweizer Krankenhaus wurden von Oktober 2019 bis Februar 2020 insgesamt 484 stationäre Patienten prospektiv in die Studie eingeschlossen. Sie erhielten schriftliche Informationen zur geteilten Anästhesiebetreuung und wurden auch persönlich darüber informiert. Der primäre Endpunkt war die Erfassung der Patientenzufriedenheit mit der geteilten Anästhesiebetreuung mittels eines validierten Fragebogens. In Gruppe 1 wurde die kontinuierliche Betreuung aus Sicht der Patienten als wichtig erachtet, aber nicht durchgeführt. In Gruppe 2 wurde eine kontinuierliche Betreuung gewährleistet. Die Patienten in Gruppe 3 wurde die kontinuierliche Betreuung als nicht wichtig betrachtet und auch nicht gewährleistet. Die Patienten in Gruppe 4 konnten sich nicht mehr daran erinnern, ob eine kontinuierliche Betreuung stattgefunden hat oder haben nicht geantwortet. Der Fragebogen wurde den Patienten nach Verlassen des Krankenhauses zugestellt.

**Ergebnisse:**

Insgesamt wurden 484 ausgefüllte Fragebögen (Rücklaufquote 81 %) ausgewertet. In Gruppe 1 (*n* = 110) betrug der durchschnittliche Unzufriedenheitsscore 25 % (95 % Konfidenzintervall [KI] 21,8–28,1), in Gruppe 2 (*n* = 61) 6,8 % (95 % KI 4,8–8,7), in Gruppe 3 (*n* = 223) 12,1 % (95 % KI 10,7–13,4) und in Gruppe 4 (*n* = 90) 15 % (95 % KI 11–18); ANOVA: *p* < 0,001, η = 0,43. Von den Patienten betrachteten 286 (59 %) die kontinuierliche Betreuung durch den gleichen Anästhesisten als eher unwichtig (34 %) oder überhaupt nicht wichtig (25 %). Für die anderen 40 % war dies wichtig (22 %) oder sehr wichtig (18 %).

**Diskussion:**

Trotz umfassender präoperativer Information über eine geteilte Anästhesiebetreuung („divided care“) betrachteten 40 % der Patienten die kontinuierliche Betreuung durch den gleichen Anästhesisten trotzdem als wichtig. Wir empfehlen die Durchführung weiterer Studien, um zu untersuchen, ob und wie die Patientenerwartung bezüglich der gängigen Praxis der geteilten Anästhesiebetreuung modifiziert und die Patientenzufriedenheit verbessert werden kann.

## Introduction and background

Patient-centered outcomes and patient-centered outcomes research are becoming increasingly important worldwide, because patient satisfaction is increasingly recognized as a very sensitive measure of a functional healthcare system [8, 12, 14, 23, 31].

From psychometrically validated surveys, we know that satisfaction, among other factors, is strongly determined by receipt of information [2, 3, 5, 6, 16, 18, 20], involvement in shared decision-making [1, 11], and continuity of care [16, 18]. From a patient’s perspective, this means that the same anesthetist handles preoperative evaluation and informed consent, intraoperative anesthesia care, and postoperative visits on the ward [24]. However, the inclusion of preoperative anesthesia units as an integral part of modern anesthesia practice and the implementation of preoperative evaluation by teleconsultation [29, 30] has promoted the “divided care” practice. This, however, might lead to patient reports of reduction in the continuity of care, and to decreased patient satisfaction [17].

Up to today, no observational study assessed patients’ expectation and perception of continuous anesthesia care and its influence on satisfaction with a psychometrically developed questionnaire. Therefore, we aimed at assessing patients’ expectation and perception of continuous care and respective impact on satisfaction.

We hypothesized that expectations of patients combined with their perceptions regarding continuous anesthesia care influences patient satisfaction. This might happen in that sense that patients who rate continuous care as not important will not be dissatisfied with divided anesthesia care.

## Material and methods

### Ethics and study design

The Medical Ethics Committee of St. Gallen, Switzerland (Business Administration System for Ethics Committees, BASEC 2017-00090) approved the study. The registration number of the study in Registry of all Projects in Switzerland (RAPS) is 2017-00090. We followed the Strengthening the Reporting of Observational Studies in Epidemiology (STROBE) reporting guidelines [10]. We obtained verbal and written informed consent from the patients.

We identified all eligible patients from our hospital (a district general hospital in Switzerland) in a consecutive order by searching our preoperative anesthesia consultation list. Patients were included if they were 16 years or older, had an American Society of Anesthesiologists (ASA) classification of 1–3, and were scheduled to undergo elective inpatient surgery in one of the following surgical disciplines: general surgery, orthopedics, gynecology, otolaryngology, urology, or plastic surgery. Patients were excluded if they had emergency surgery, insufficient knowledge of the German language, or cognitive deficits.

All patients were sent an informative brochure describing the process of anesthesia care, including preoperative evaluation. The patients who read the brochure were thus already informed that for organizational reasons it was unlikely that the preoperative interview and anesthesia care would be provided by the same anesthetist. A face-to-face preoperative evaluation was performed at the preoperative anesthesia ward, and consent for anesthesia was obtained. At this time, patients were again informed about the possibility of divided anesthesia care and about the fact that the anesthetic would very likely be provided by a different anesthetist due to organizational reasons. They were also assured that all information, including the discussed and agreed upon anaesthetic technique would be available at the anesthesia appointment on the day before surgery as well as on the day of surgery.

### Assessment of patient satisfaction

Patients were informed that they would receive a patient satisfaction questionnaire 1–2 weeks after discharge (to reduce social desirability bias which describes a tendency to answer questions as expected) [25]. If no response was received after 2 weeks, a reminder questionnaire was mailed. To improve the response rate, a personalized cover letter from the department, assuring anonymity, and a prepaid return envelope were delivered to the patient. All questionnaires were sent to a statistical institute for analysis.

#### Questionnaire

The questionnaire, which particularly addressed patient satisfaction in anesthesia care, had been developed together with the Picker Institute, an institute specializing in surveys on patient satisfaction in general. The instrument had been psychometrically developed and tested for content and construct validity and internal consistency when it was first used from 2000 to 2002 [16]. In the meantime, this instrument has been used and revalidated in several studies [17, 24, 25]. The questionnaire consists of 55 items covering all aspects of anesthesia care. Of these 55 questions, 29 were designed as quality or problem questions, each of them addressing one specific aspect of quality to be answered by the surveyed patients (Supplement 1). The other 26 questions ask for structural data about patient treatment within anesthesia care and are not applied in the analysis of patient satisfaction (e.g. side effects, subjective state of health). If a quality question was answered partially or entirely negatively, this was considered a deficit in anesthesia care. According to the psychometric validation study the 29 problem questions were grouped into 6 categories, also referred to as dimensions: “information/involvement in decision-making”, “respect/confidence”, “delays”, “nursing care in recovery room”, “continuity of personal care by anesthetist”, and “pain management” [16]. The percentage of deficits mentioned in the items belonging to every dimension was defined as the problem or dissatisfaction score for each of the 6 dimensions, ranging from 0 to 100%. The mean of the problem scores for all 29 problem questions defined the total problem score (dissatisfaction score), also with possible values between 0% (none of the 29 items rated as problematic) and 100% (all quality items considered unsatisfactory).

The focus of this study was the dimension “continuity of care by the same anesthetist” (Table 1).

**Table 1:** Items including the dimension “continuity of care by the same anesthetist”

“Did you receive information from an anesthetist about your upcoming anesthesia?”
“Did you know which anesthetist would conduct your anesthesia?”
“Did the same anesthetist who informed you conduct your anesthesia?”
“Did the same anesthetist visit you after the operation?”

We linked the answers to two questions: “did the same anesthetist who performed your preoperative evaluation conduct your anesthesia?” and “how important do you rate receiving your anesthetic from the same anesthetist who conducted your preoperative evaluation?”.

It was defined as a problem if patients who considered the above mentioned question to be “very important” or “rather important” (taking those two answer categories together) did not have continuous care (i.e., with the same anesthetist conducting the preoperative evaluation and the anesthesia). On the other hand, we considered it to be no problem if the patient received continuity of anesthesia care, or if multiple people provided anesthesia care but the patient stated that continuity of care by the same anesthetist was “rather unimportant” or “not important at all”.

#### Outcome parameters

The primary outcome was the assessment of the mean total problem score (dissatisfaction score). We divided the patients into four groups: in group 1, continuity of care by the same anesthetist was considered “very important” or “rather important” but the anesthetist performing anesthesia was not the same. In group 2, the same anesthetist conducted the preoperative evaluation and performed anesthesia. In group 3, continuity of care by the same anesthetist was regarded as “rather unimportant” or “not important at all” and care was not performed by the same anesthetist. In group 4, patients could not remember whether their anesthetic care was provided by the same anesthetist or did not answer. Secondary outcomes were the problem scores of each underlying dimensions.

Data on age, sex and ASA classification were available for all 739 patients assessed for eligibility. Reasons for non-enrolment were documented for the 139 patients who were excluded. For all 600 patients recruited to the study, hospital data on the following sociodemographic and clinical characteristics were available: age, sex, body mass index (BMI), length of hospital stay, number of hospital stays in the last 6 months, ASA classification, anesthetist who performed the preoperative evaluation (consultant or resident), and anesthetist who performed anesthesia (consultant or resident), days between preoperative evaluation and surgery, type of anesthesia (general anesthesia, regional anesthesia, monitored anesthesia care), surgical discipline, extent of surgery (minor, moderate, major), and complications (Clavien-Dindo classification) [9].

From the questionnaire, we analyzed the following data: age, sex, educational level (primary/basic school, secondary/comprehensive school, vocational school, high school, or college/university), type of insurance (standard, semi-private, or private), and self-rated health.

We assessed potential correlations of these parameters with the recruiting rate and response rate, indicating potential selection bias.

We also analyzed how these parameters related to satisfaction parameters.

The recruitment rate was defined as the percentage of eligible patients who were recruited into the study, and the response rate was defined as the percentage of recruited patients who returned completed questionnaires.

### Statistical analysis

Statistical analysis included univariate analyses as well as crosstabs (including χ^2^ and Cramer’s V) and analysis of variance (ANOVA, η). With more than two subgroups to be compared, the multiple comparison of means was applied (Scheffé test). When appropriate, correlation analysis was performed. Internal consistency of the six quality dimensions was re-assessed by applying reliability analysis. In all analyses, *p* < 0.05 (two-tailed) was treated as the threshold for significance.

#### Sample size calculation

We (a priori) aimed to recruit a minimum of 300 study participants with complete data for the analysis. With 300 cases, the 95% confidence interval (CI) [two-sided] for a proportion value of 80% (commonly used for satisfaction items) would be restricted roughly within ± 5% limits (75.1–84.1%) [22].

According to our previous experience with patient satisfaction surveys, we expected a participation rate of approximately 65% [4, 11, 24, 25]. We therefore planned to enrol 600 patients in the study.

All analyses were performed using SPSS 25 (IBM, Chicago, IL, USA).

## Results

**Fig. 1:**
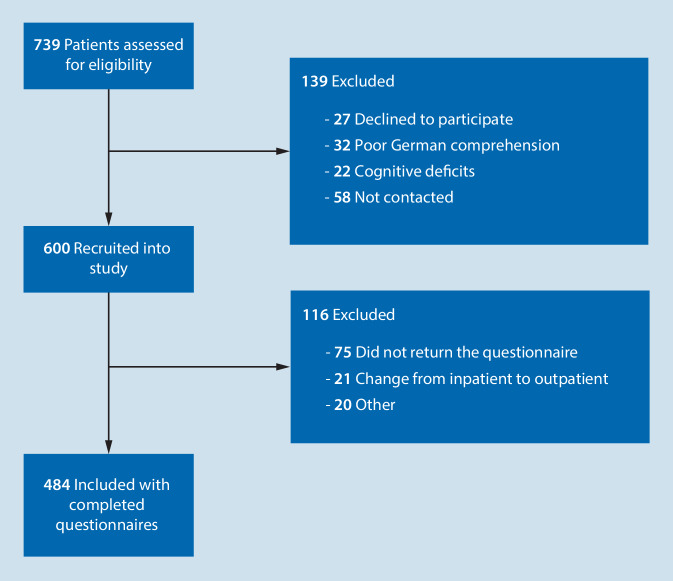
Study flow chart

From October 2019 to February 2020, 484 patients were included in the analysis. The study flow diagram is shown in Fig. 1. The final cohort comprised 484 completed questionnaires. Mean age of the 484 patients was 55.8 years (standard deviation 16.8). There were 228 men (47%) and 245 (51%) women; 11 patients (2%) did not respond.

Of the 739 patients who were assessed for eligibility, 27 declined to participate, 32 were excluded due to poor German comprehension, 22 were excluded due to cognitive deficits and 58 were not contacted, mainly because they were admitted directly to the ward and therefore did not appear on the hospital’s preoperative admission list.

Of the 600 patients who were recruited into the study, 75 did not return the questionnaire, 21 changed to outpatient status, and 20 were excluded for other reasons. For analysis, 484 completed questionnaires were included.

The total recruitment rate was 81% (600 out of 739). The reasons for non-recruitment are given in Fig. 1.

No significant sex-related differences between the 600 patients recruited and the 139 patients excluded were found (data not shown). According to ASA classification, patients with higher values had a significantly greater chance of not being recruited (ASA classification 1: 9.1%, ASA classification 2: 19.8%, ASA classification 3: 36.4% excluded; *p* < 0.001). Patients with higher ASA classification were also older (59.1 years versus 54.9 years; *p* = 0.012) and had more cognitive deficits. Thus, those patients not able to take part in the study were on average older and had a higher burden of disease, in terms of ASA classification and cognitive deficits.

The response rate (reminder included) was 81% in total (484 out of 600). The reasons for exclusion are given in Fig. 1. The response rate was significantly higher in older patients (86% in patients ≥ 55 years vs. 74% in patients < 55 years; χ^2^ = 13.99, df = 1, *p* < 0.001), and in women (84% vs. 77%; χ^2^ = 3.95, df = 1, *p* = 0.047). The response rate in patients who underwent major surgery was significantly higher (minor surgery 64%, moderate 80%, major 93%; η = 0.24, *p* < 0.001). For type of anesthesia a significantly lower response rate in the very small “monitored anesthesia care” group was found: only four of nine persons in this group filled out the questionnaire (87% were patients receiving regional anesthesia and 81% were patients receiving general anesthesia; η = 0.13, *p* = 0.006). There was no significant influence on response rate for all other parameters taken from hospital data, i.e., BMI, length of stay, number of hospital stays in last 6 months, ASA classification (with 73% lower in ASA classification 3, but not significant), anesthetist who performed the preoperative evaluation and anesthetist who provided anesthesia for the surgery, days between preoperative evaluation and surgery, surgical discipline, extent of surgery, or complications.

Hospital data, including the baseline characteristics between responding patients (patients with completed questionnaires) and non-responding patients (recruited patients), are shown in Tables 2 and 3.

**Table 2:** Hospital data, including the baseline characteristics between patients with completed questionnaires and recruited patients

Hospital data				
		Patients with completed questionnaires (*n* = 484)	Recruited patients (*n* = 600)	*p*-value
Age, two groups	< 55 years	199 (41)	269 (45)	*p* < 0.001*
	≥ 55 years	285 (59)	331 (55)	
Sex	Female	250 (52)	298 (50)	*p* = 0.047*
	Male	234 (48)	302 (50)	
BMI^a^	< 20	22 (5)	29 (5)	*p* = 0.750
	20–< 25	153 (32)	193 (32)	
	25–< 30	172 (36)	215 (36)	
	30–< 35	99 (20)	118 (20)	
	≥ 35	38 (8)	45 (8)	
Number of (further) hospital admissions in last 6 months	None	418 (86)	519 (87)	*p* = 0.323
	1	57 (12)	72 (12)	
	2 and more	9 (2)	9 (2)	
ASA^b^ classification	1	136 (28)	170 (28)	*p* = 0.228
	2	312 (64)	380 (63)	
	3	36 (7)	50 (8)	
Amount of time between preoperative evaluation and surgery	1 day	85 (18)	269 (45)	*p* = 0.635
	1 day–1 week	176 (36)	215 (36)	
	> 1 week	223 (46)	108 (18)	
Preoperative evaluation	Resident	223 (46)	276 (46)	*p* = 0.941
	Consultant	261 (54)	324 (54)	
Performance of anesthesia	Resident	136 (28)	173 (29)	*p* = 0.204
	Consultant	348 (72)	419 (71)	
Type of anesthesia	General anesthesia	377 (78)	464 (78)	*p* = 0.006*
	Regional anesthesia	103 (21)	119 (20)	
	Monitored anesthesia care	4 (1)	9 (2)	
Extent of surgery	Minor	52 (11)	81 (14)	*p* < 0.001*
	Moderate	265 (55)	332 (56)	
	Major	167 (35)	179 (30)	
Surgical discipline	General surgery	115 (24)	149 (25)	*p* = 0.537
	Orthopedics	191 (39)	228 (39)	
	Gynecology	62 (13)	75 (13)	
	Otolaryngology and maxillofacial surgery	64 (13)	78 (13)	
	Urology	46 (10)	56 (9)	
	Plastic surgery	6 (1)	6 (1)	
Complications (Clavien-Dindo classification)	< 3	477 (99)	581 (98)	*p* = 0.117
	≥ 3	7 (1)	11 (2)	

Data in brackets indicate the percentage of patients in each of the groups (%). *p* < 0.05 (*) [χ^2^] marks statistically significant values

^a^ Body mass index

^b^ American Society of Anesthesiologists

**Table 3:** Data of patients with completed questionnaires

Questionnaire data		
		Patients with completed questionnaires (*n* = 484)
Age (standard deviation)	–	Mean = 55.8 years (16.8)
Age, two groups (%)	< 55 years	197 (41)
	≥ 55 years	270 (56)
	No answer	17 (4)
Gender (%)	Female	245 (51)
	Male	228 (47)
	No answer	11 (2)
Education (%)	Primary or secondary school	85 (18)
	Vocational school	228 (47)
	University	148 (31)
	No answer	23 (5)
Type of insurance (%)	General	299 (62)
	Semi-private	107 (22)
	Private	68 (14)
	No answer	10 (2)
Self-rated health (%)	Excellent	90 (19)
	Very good	191 (39)
	Good	156 (32)
	Middle	32 (7)
	Poor	5 (1)
	No answer	10 (2)
Number of days in hospital (%)	1–3 days	307 (63)
	4–6 days	129 (27)
	1–3 weeks	32 (7)
	> 3 weeks	3 (1)
	No answer	13 (3)

### Patient satisfaction

A total of 286 patients (59%) assessed continuity of care by the same anesthetist as rather unimportant (*n* = 166, 34%) or not important at all (*n* = 120, 25%). The other 40%, however, appraised continuity of care by the same anesthetist as important (*n* = 104, 21%) or very important (*n* = 89, 18%), five people did not answer.

**Fig. 2:**
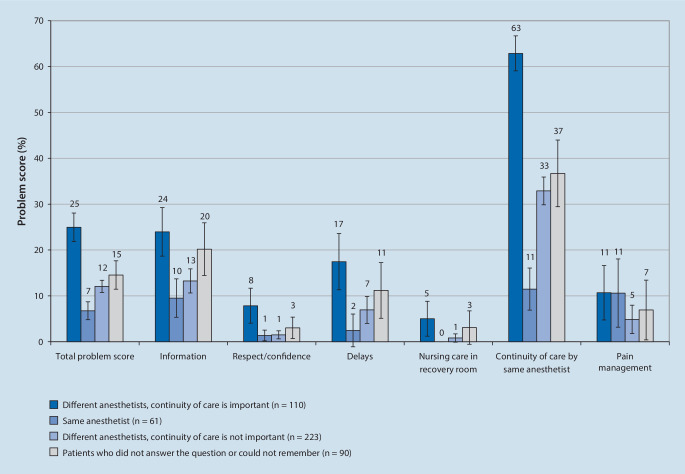
Mean total problem score and problem scores of the dimensions 1–6 (*bars* indicate 95% CI)

In group 1 (*n* = 110), continuity of care was considered important, but was not received, leading to a mean total dissatisfaction score of 25% (95% CI 22–28). In group 2 (*n* = 61), continuity of care was performed, leading to a mean score of 7% (95% CI 5–9). In group 3 (*n* = 223), continuity of care was not considered important and was not received, with a mean score of 12.1% (95% CI 10.7–13.4). In group 4 (*n* = 90), patients had no memory or did not answer, leading to a mean score of 15% (95% CI 11–18) (Fig. 2). These differences were significant in the ANOVA, with* p* < 0.001 and η = 0.43. In the multiple comparison of means (Scheffé test), group 1 was significantly higher than all other groups (at least *p* < 0.05); whereas group 2 was significantly lower.

The four groups did not differ significantly in any of the questionnaire or hospital parameters in terms of the sociodemographic or clinical characteristics mentioned above.

Patients who rated provision of care by the same anesthetist at the preoperative evaluation and during surgery as “very important” or “rather important”, but who did not have the same anesthetist (group 1), were much more dissatisfied with dimension number 5: “continuity of personal care by the anesthetist” (63% vs. 11%, 33%, and 37%, respectively in the groups 2, 3 and 4, ANOVA: *p* < 0.001, η = 0.54, Scheffé test: *p* < 0.001 for group 1 versus all other groups and *p* < 0.001 for group 2 versus all other groups).

Furthermore, significant differences were found between group 1 (highest dissatisfaction levels) and groups 2–4 for dimensions 1, “information/involvement in decision-making”, 2, “respect/confidence”, and 3, “delays”. For dimensions 4, “nursing care in recovery room” and 6, “pain management”, no significant differences were found (Fig. 2).

The mean total dissatisfaction score based on 29 quality questions of all 484 patients was 15% (CI 13.6–16.0).

One of the four questions used to assess continuity of anesthesia care involved continuity from preoperative evaluation to surgery: “did the same anesthetist who informed you conduct your anesthesia?” 61 patients (13%) answered with yes, 336 answered with no (69%), 83 patients said that they could not remember (17%); two people did not answer, and two people stated (falsely) that they had no preoperative interview.

## Discussion

We found that despite receiving comprehensive preoperative information about divided anesthesia care, a large number of patients still considered continuity of care by the same anesthetist important. In this group, other aspects of satisfaction with anesthesia care and hence total patient satisfaction were significantly worse if continuity could not be ensured.

It is not surprising that patients who say they value continuity of care were dissatisfied when it was not delivered. However, even if the result is relatively obvious, we have learned at least three things that are worth calling attention to. First, despite receiving comprehensive preoperative information about divided anesthesia care, and despite the fact that divided care is common practice worldwide, the number of patients who considered continuity of care to be important in our setting remained high (40%). Second, those patients who did not receive continuity of care were significantly more dissatisfied in most other dimensions as well, compared to the other groups. A possible explanation could be that these patients might have a more critical attitude per se and might have been dissatisfied regardless of continuity of care. The fact that there was a significant difference in dissatisfaction in the dimensions of information/involvement in decision-making and respect/confidence also supports this notion. Third, even in those patients who consider continuity of care not important, more than 12% were dissatisfied with total anesthesia care.

Our results are in contrast to those of other researchers who found that patient satisfaction with anesthesia care did not depend on whether patients received continued anesthesia care, even though a large proportion of responders felt that continuity of care was “very important” or even “essential” [15]. One explanation might be that we used a psychometrically developed questionnaire including all aspects of satisfaction with anesthesia care [28]. Surveys that simply assess overall satisfaction “were you satisfied with your anesthesia*?*” show that most patients are satisfied [19]. However, this is an overly optimistic picture. In other words, “surveys which are less than excellent should give rise to concern” [7]. A further reason for the discrepancy could be that we sent the instrument to patients after hospital discharge. In-hospital surveys, as conducted by other researchers, risk incorporating social desirability bias, which means that patients tend to assess the care they are receiving more positively, since they are still “under care” and might fear negative consequences [13, 26].

Our findings reveal a dilemma concerning preoperative evaluation. On the one hand, implementation of preoperative anesthesia wards is an inherent part of modern anesthesia practice (divided care), and it is very unlikely that this direction will change. On the contrary, preoperative evaluation by teleconsultation will probably strengthen this in the near future [29, 30]. Nevertheless, we are confronted with the fact that a considerable number of patients, in our case 40%, still assess continuity of care by the same physician as important [27]. Certainly, as the total problem score, including all patients in our setting, was only 15%, it could be argued that this is rather a luxury problem than a worthwhile issue. However, looking at those patients in our study who regarded continuity of care as important but did not receive it, we learned that they were significantly more dissatisfied in other dimensions of anesthesia care as well.

Realistically, we cannot completely solve this dilemma. The crux is to identify those patients who are more or less fixated on continuity of care and, if at all possible, try to ensure it.

However, based on our results, it is impossible to estimate this preference for continued care a priori, based on sociodemographic and hospital data (age, sex, ASA classification, etc.), since these parameters were randomly distributed between the groups.

Based on the surprisingly high dissatisfaction scores in group one, it appears that a further randomized study might shed light on whether there are alternative ways to modify patient expectations regarding the (un)importance of continuity of anesthesia care without leading to a lack of quality, and hence increase patient satisfaction.

### Limitations

Our study has several limitations. It was not randomized for continuity of care by the same anesthetist. We carefully considered this issue as we designed the study and decided against randomization for two reasons. First, practically, it would have been almost impossible to adapt this for daily routine, and hence data quality could not have been guaranteed. Second, today’s anesthesia departments are primarily organized as divided care facilities. It would therefore make no sense to look at a process that does not reflect reality any more.

In addition, randomization to address whether patients were informed about divided care (e.g., face-to-face vs. brochure only) would not have been justified either. We already know from previous studies that being informed is very important for patient satisfaction with anesthesia care. From an ethical point of view, withholding information would at the very least be debatable. In addition, from a legal perspective, we are required to inform our patients in detail.

Second, external validity has to be questioned, as the results of a population in a medium-sized district hospital may not be generalizable to other settings. The strict methodology, including unbiased sampling and a high response rate, however, ensures that a certain generalizability can be assured [21].

In summary, despite receiving comprehensive preoperative information about divided anesthesia care, 40% of patients still considered continuity of care by the same anesthetist important. We recommend further research evaluating whether and how patient expectations can be modified and patient satisfaction with divided anesthesia care can be increased.

## Supplementary Information

**Supplement 1: **Quality (problem) questions

## Declarations

All procedures performed in studies involving human participants or on human tissue were in accordance with the ethical standards of the institutional and/or national research committee and with the 1975 Helsinki declaration and its later amendments or comparable ethical standards. Informed consent was obtained from all individual participants included in the study.
