# Supplementary material for: Patient satisfaction with divided anesthesia care
Source: Anaesthesiologie. 2022 Aug 29;72(2):97–105. doi: 10.1007/s00101-022-01192-x (PMC9892151; doi:10.1007/s00101-022-01192-x)
Supplement: Supplementary file 1 — Supplement 1: Quality (problem) questions [file 101_2022_1192_MOESM1_ESM.docx]

**Quality (problem) questions**

**Information / Involvement in decision-making**

Overall, did you feel that you received enough information about your upcoming anaesthesia before you entered the hospital?

Were you able to talk to the anaesthetist about any fears or concerns you had related to the upcoming anaesthesia?

If you asked your anaesthetist questions about the upcoming anaesthesia at this meeting, did you get answers you could understand?

Did you feel that you had enough say in the choice of anaesthetic method?

Did the anaesthetist tell you how you would feel after the anaesthesia?

Did you feel that the anaesthetist took enough time to talk with you?

Did you have enough privacy during this informational interview?

When the anaesthesia was given, did the team explain what was happening?

Did the anaesthesia team (anaesthetist and nurse anaesthetist) explain what was happening?

**Respect / Confidence**

Did you have confidence in the anaesthetist?

How would you rate the courtesy of your anaesthetist?

Did you feel your anaesthetist took you seriously?

Did you have confidence in the nurse anaesthetist?

How would you rate the courtesy of your nurse anaesthetist?

Did you feel your nurse anaesthetist took you seriously?

**Delays**

Were you taken to the operating room at the scheduled time?

Were you given a reason for the delay?

Was the anaesthesia started on time?

Did anyone tell you the reason for the delay?

**Nursing care in recovery room**

How would you rate the availability of the nursing staff in the recovery room?

How would you rate the politeness of the nursing staff in the recovery room?

**Continuity of personal care by anaesthetist**

When you were in the hospital, did an anaesthetist stop by to discuss the upcoming anaesthesia with you before your surgery?

Did you know which anaesthetist would be performing your anaesthesia?

Was the anaesthetist the same person who came to your informational interview?

Which member of the anaesthesia team visited you on the ward after the operation?

**Pain management**

When the anaesthesia was given, did the team address your pain?

Did the anaesthesia team address your pain during the operation?

Did the nursing personnel in the recovery room do everything possible to address your pain?

Did the hospital staff do everything possible to address your pain?
